# Frontal theta oscillations during emotion regulation in people with borderline personality disorder

**DOI:** 10.1192/bjo.2024.17

**Published:** 2024-03-04

**Authors:** Moritz Haaf, Nenad Polomac, Ana Starcevic, Marvin Lack, Stefanie Kellner, Anna-Lena Dohrmann, Ulrike Fuger, Saskia Steinmann, Jonas Rauh, Guido Nolte, Christoph Mulert, Gregor Leicht

**Affiliations:** Department of Psychiatry and Psychotherapy, Psychiatry Neuroimaging Branch, University Medical Centre Hamburg-Eppendorf, Hamburg, Germany; Department of Neurophysiology and Pathophysiology, University Medical Centre Hamburg-Eppendorf, Hamburg, Germany; Department of Psychiatry and Psychotherapy, Psychiatry Neuroimaging Branch, University Medical Centre Hamburg-Eppendorf, Hamburg, Germany; and Centre for Psychiatry and Psychotherapy, Justus Liebig University, Giessen, Germany

**Keywords:** Borderline personality disorder, emotionally unstable personality disorder, EEG, cognitive reappraisal, prefrontal cortex

## Abstract

**Background:**

Borderline personality disorder (BPD) is a severe psychiatric disorder conceptualised as a disorder of emotion regulation. Emotion regulation has been linked to a frontolimbic network comprising the dorsolateral prefrontal cortex and the amygdala, which apparently synchronises its activity via oscillatory coupling in the theta frequency range.

**Aims:**

To analyse whether there are distinct differences in theta oscillatory coupling in frontal brain regions between individuals with BPD and matched controls during emotion regulation by cognitive reappraisal.

**Method:**

Electroencephalogram (EEG) recordings were performed in 25 women diagnosed with BPD and 25 matched controls during a cognitive reappraisal task in which participants were instructed to downregulate negative emotions evoked by aversive visual stimuli. Between- and within-group time–frequency analyses were conducted to analyse regulation-associated theta activity (3.5–8.5 Hz).

**Results:**

Oscillatory theta activity differed between the participants with BPD and matched controls during cognitive reappraisal. Regulation-associated theta increases were lower in frontal regions in the BPD cohort compared with matched controls. Functional connectivity analysis for regulation-associated changes in the theta frequency band revealed a lower multivariate interaction measure (MIM) increase in frontal brain regions in persons with BPD compared with matched controls.

**Conclusions:**

Our findings support the notion of alterations in a frontal theta network in BPD, which may be underlying core symptoms of the disorder such as deficits in emotion regulation. The results add to the growing body of evidence for altered oscillatory brain dynamics in psychiatric populations, which might be investigated as individualised treatment targets using non-invasive stimulation methods.

Borderline personality disorder (BPD) is a severe psychiatric disorder with a prevalence in the general population of 1–3%.^1^ It is characterised by pervasive patterns of emotional sensitivity, affective lability, instability in self-image as well as in interpersonal relationships, and a deficit of appropriate regulation strategies.^2^ BPD is therefore conceptualised as a disorder of emotion regulation. According to Linehan's biosocial model, individuals diagnosed with BPD show an increased emotional sensitivity from birth, with an inability to regulate intense emotional responses, which makes these individuals particularly vulnerable to experiencing negative affect and to engaging in dysregulated behaviours in an attempt to manage these affects.^2,3^ Individuals diagnosed with BPD often describe navigating a spectrum of intense emotions, which frequently leaves them feeling overwhelmed and uncertain about managing these intense feelings. On numerous occasions, this emotional turmoil compels them to seek relief from emotions they struggle to express. These individuals report that, at times, their quest for emotional respite drives them to adopt detrimental coping mechanisms. They tend to lean on easily accessible or straightforward approaches to disengage themselves emotionally.^4^

## Emotion regulation and its neural basis

The neural basis of emotion regulation has attracted a lot of attention over recent decades, as emotion dysregulation is a core symptom of various psychiatric disorders.^5,6^ The limbic system has generally been linked to emotion generation and detection, whereas cognitive control processes are associated with prefrontal brain areas such as the ventromedial and lateral prefrontal cortices and the parietal cortices.^5,7^

Cognitive reappraisal is one treatment strategy that aims to enable patients to better control their emotions by changing the emotional impact of an event by reinterpreting its meaning. Neuroimaging studies consistently highlight the involvement of specific prefrontal regions (dorsomedial, dorsolateral and ventrolateral prefrontal cortex – dmPFC, dlPFC and vlPFC respectively) and the amygdala in cognitive reappraisal tasks.^6^ However, there is some controversy regarding the exact interplay of these brain regions during these tasks.^8^ One proposition is that the prefrontal areas modulate emotional responses by exerting regulatory control over the amygdala, but the dynamics of this interaction are subject to ongoing research and debate.^6,8^

## Neural correlates of emotion regulation in people with BPD

As emotion regulation plays an important role not only in the pathogenesis of BPD but also in its treatment, numerous neuroimaging studies have investigated the neural correlates of emotion regulation in individuals diagnosed with BPD. These studies revealed structural and functional regions involved in the dysregulation of emotions, suggesting an altered frontolimbic inhibitory network.^9–11^ In individuals with BPD, an increased activation of the amygdala as part of the limbic system could be found in the processing of negative emotional stimuli, whereas the recruitment of frontal brain regions (e.g. dlPFC and vlPFC) is blunted.^9,10^ Thus, reduced frontal inhibition during emotion regulation processes seems to play an important – yet not fully understood – role in the pathophysiology of BPD.^10,12^

## Theta oscillations as a neurophysiological correlate of emotion regulation

Although functional magnetic resonance imaging (fMRI) provides invaluable insights into the brain regions involved in emotion regulation, the high temporal resolution of electroencephalogram (EEG) recordings can significantly enhance our understanding of the underlying neurophysiological mechanisms.^13^ The millisecond-range resolution of EEG is particularly adept at capturing the brain's oscillatory activities, a field that has garnered increasing interest. Investigating these oscillations sheds light on the functional network dynamics within the brain, as it is believed that the synchronised firing of neurons in different brain regions is key to their communication.^14^ Previous basic research on the connectivity between the amygdala and prefrontal regions showed increased oscillatory coupling in the theta frequency range in mice in fear conditioning and fear extinction processes.^15^

In addition, various studies underscore the important role of low-frequency oscillations in the theta band in the context of emotion regulation,^16–18^ but also in the context of cognition and perception.^19^

Ertl et al^16^ investigated the role of frontal theta oscillations in emotion regulation in healthy volunteers and showed increased theta activity during cognitive reappraisal tasks as well as a correlation between the strength of theta power and self-reported regulation success. Scalp EEG limitations in detecting cortical–subcortical interactions led to using self-reported affect ratings as proxies for amygdala activation in emotion regulation, a method debated in recent research. On the one hand, it has been reported that self-reports of regulation success are associated with amygdala activation.^20^ However, on the other, research^21^ highlights the amygdala's broader role beyond mere emotion processing, questioning the accuracy of self-reports in fully capturing amygdala activity in emotional contexts.

## Aims and hypotheses

The aim of this study was to investigate whether people with BPD show different patterns in theta oscillations during emotion regulation in a cognitive reappraisal task. Based on findings in previous neuroimaging studies we hypothesised that individuals diagnosed with BPD face challenges in effectively regulating negative affect, resulting in reduced oscillatory coupling in the theta band compared with matched controls. Regulation success was expected to be positively correlated with the strength of the theta activity.

## Method

### Participants

A sample of 25 women assigned a diagnosis of BPD (mean age: 27.2 years, s.d. = 6.3 years) and the same number of matched controls were recruited for the study. The matching was based on sex (assigned at birth), age (mean 25.7 years, s.d. = 5.6 years), education and handedness (24 right-handed participants in each group).

Eligibility criteria for all participants excluded those currently experiencing substance use challenges or significant physical or neurological health conditions. For individuals in the control group, additional eligibility criteria excluded those with any history of receiving psychiatric care or a family history of mental health diagnoses. Eligibility was determined through a comprehensive interview led by an experienced clinical psychiatrist or psychologist with substantial clinical background. For participants experiencing traits associated with BPD, a diagnostic assessment was conducted using the Structured Clinical Interview for DSM-IV Axis II Personality Disorders (SCID-II).^22^ To gauge the emotional well-being of the participants, the Montgomery–Åsberg Rating Scale for Depression (MADRS) was utilised.^23^ Inclusion in the study required a MADRS score below 10, indicative of, at most, mild depression. Matched controls were recruited from the community through advertisement and word of mouth. The authors assert that all procedures contributing to this work comply with the ethical standards of the relevant national and institutional committees on human experimentation and with the Helsinki Declaration of 1975, as revised in 2008. All procedures involving human participants were approved by the Ethical Committee of the Medical Association Hamburg (PV4009). Written informed consent was obtained from all participants.

### Stimuli

As stimuli, 100 negative and 50 neutral pictures were selected from the International Affective Picture System.^24^ Pictures were not repeated throughout the experiment. The negative pictures showed scenes such as accidents, injuries or assault, whereas the neutral pictures showed scenes such as everyday objects or people in common life situations.

### Emotion regulation strategy

The concept of cognitive reappraisal strategies in emotion regulation was introduced to the participants prior to the experiment. The standardised instruction for cognitive reappraisal given to each participant was the German translation of the instructions used in various other studies.^25–27^ Participants were asked to reappraise any negative pictures in a positive way. They were instructed to go beyond the apparent or surface meaning of depicted events and to reconsider the possible antecedents, outcomes and/or reality of the events. After a training set, when the participants reported that they found an efficient emotion regulation strategy, the main experiment began.

### Paradigm

**Fig. 1:**
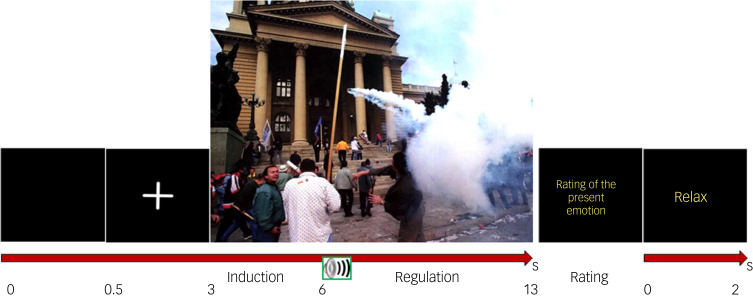
The sequence of the cognitive reappraisal task. The trial started with a black screen presented for 500 ms, followed by presentation of a fixation cross for 2.5 s. The fixation cross was used to reduce eye movements and focus the gaze at the centre of the screen for picture onset. Thereafter, the picture was presented for 10 s. Three seconds after picture onset a digitised human voice gave a one-word regulation instruction ‘*verringern*’ (Engl. decrease) or ‘*fortfahren*’ (Engl. maintain), marking the beginning of the emotion regulation phase of the trial. An emotion rating scale was then presented on the screen, with extremes marked with ‘negative’ (maximum rating value 100) and ‘neutral’ (minimum rating value 0) and participants were instructed to rate their current emotion by moving a bar on the scale using a computer mouse. After pressing the mouse button, the word ‘relax’ appeared on the screen for 2 s and the trial ended.

The paradigm is a modified version of the one used in a previous study by our group.^16^ Three conditions were created: the neutral condition with presentation of a neutral picture followed by the regulation instruction ‘*fortfahren*’ (Engl. maintain), the maintenance condition with presentation of a negative picture followed by the regulation instruction ‘*fortfahren*’ (Engl. maintain) and the reappraisal condition with presentation of a negative picture followed by the regulation instruction ‘*verringern*’ (Engl. decrease), which prompted the participants to apply their emotion regulation strategy. Participants were instructed to let themselves react to the depicted scene as they naturally would whenever they heard the instruction ‘*fortfahren*’ (Engl. maintain). Thereafter, the participants were instructed to rate their current emotion using a visual emotion rating scale presented on the screen, with extremes marked as ‘negative’ (maximum score, 100) and ‘neutral’ (minimum, 0). The sequence of a trial is depicted in Fig. 1 and a description can be found in the Supplementary material available at https://doi.org/10.1192/bjo.2024.17.

The experiment consisted of three experimental blocks with a break of 5 min between the blocks. The entire experiment took about 60 min. Each of the three experimental blocks consisted of 50 trials with a pseudo-randomised order of conditions.

### Questionnaires

Prior to the EEG recording, each participant filled in a German version of the Emotion Regulation Questionnaire (ERQ).^28^ Participants’ handedness was assessed by means of the Edinburgh Handedness Inventory.^29^

### EEG acquisition

The EEG recording took place in a sound-attenuated and electrically shielded room. Participants were seated on a slightly reclined chair facing a 19-inch computer monitor. The distance between the participants’ eyes and the monitor was approximately 1 m. Instructions were presented via headphones. Continuous EEG was recorded using Ag/AgCl electrodes mounted in a 64-channel actiCAP system (Brain Products, Gilching, Germany) and BrainVision Recorder® software for Windows, version 1.20 (Brain Products). Electrodes were positioned in an extended 10/20 system with FCz as the reference electrode. The impedances were always kept below 5 kΩ.

### EEG pre-processing

EEG data analysis was conducted in Matlab for Windows (MathWorks®) using the open-source toolbox Fieldtrip.^30^ EEG data were segmented into 13 s epochs starting 6 s prior to the instruction, filtered with a low-pass filter (100 Hz), a high-pass filter (0.3 Hz) and a band-stop filter for line noise (50 Hz), detrended and demeaned. Thereafter, noisy channels were interpolated.

Trials containing prominent muscle and movement artifacts were semi-automatically detected and rejected from further analysis. After re-referencing to a common average reference, eye movements and blink artifacts were corrected using independent component analysis (ICA) on appended trials.

Ratings of emotions were averaged for each participant and condition. Ratings of emotions for one participant were not acquired because of technical problems. Given that the EEG data had sufficient quality, this participant was excluded only for analyses involving the ratings of emotions.

### Time–frequency analysis of induced activity

The trial-averaged event-related potential for each condition (time-locked to the onset of the regulation instruction) was subtracted from the corresponding pre-processed single-trial segments to obtain the induced activity. Next, time–frequency analysis was performed on these data using a sliding Hanning window with a 50 ms slide. The length of the time window for time–frequency analysis was set to 2 s for each frequency between 1 and 30 Hz (0.5 Hz steps). Subsequently, frequency-wise baseline correction (subtraction) was applied using a baseline period from 1.2 to 0.2 s prior to picture onset. Averages were calculated for each participant and condition separately, and these were used for calculation of grand averages for each group and condition.

To compute the regulation-associated theta activity, the individual mean activity in the maintenance condition was subtracted from the individual mean activity in the reappraisal condition. Based on previously published results^16–18^ our analysis focused on regulation-associated theta activity (3.5–8.5 Hz) following the regulation instruction (1–5 s).

Differences in regulation-associated theta activity in the participants with BPD compared with the matched controls were tested using a non-parametric, permutation-based statistical approach using the MATLAB toolbox FieldTrip, which incorporates spatial clustering for multiple comparisons correction.

Initially, the two-sided independent-samples *t*-statistics for each electrode comparing the two groups are calculated. Electrodes showing a preliminary indication of significant differences (α < 0.05) are then considered for cluster formation. Spatial clusters are formed by grouping these neighbouring electrodes. The sum of the *t*-statistics within each cluster is calculated to represent the cluster-level statistic.

The formation of these initial clusters is followed by permutation testing involving 100 000 randomisations. In each permutation, data labels are randomly shuffled and the entire process of calculating *t*-statistics, identifying significant electrodes, forming clusters and summing their *t*-statistics is repeated. This generates a distribution of cluster-level statistics under the null hypothesis of no group difference. The *P*-value of the initial cluster is derived from this distribution by determining the proportion of permutations in which the recalculated cluster-level statistic was as extreme as or more extreme than the observed initial cluster. Adjusting this proportion to account for the total number of permutations provides the *P*-value.

### eLORETA source localisation of the theta power

The pre-processed single trials of induced theta activity were re-segmented to the regulation phase (1–5 s after the regulation instruction) and to the baseline period (1.2–0.2 s prior to picture onset). A subsequent fast Fourier transformation was performed using a Hanning taper with 0.5 Hz steps from 3.5–8.5 Hz (with data zero-padded to the onset of the regulation instruction). On the averages of these complex data, the exact low-resolution brain electromagnetic tomography (eLORETA)^31^ algorithm (as implemented in the METH toolbox) was applied for each participant to calculate source power values for each voxel in the source grid (2839 voxels located on the cortical surface). These values were then normalised for each voxel by dividing them by the respective baseline period power values, thus obtaining a theta power ratio. To explore the regulation-associated variations in the theta power ratio, a voxel-wise comparison was conducted. This involved subtracting the theta power ratio of the maintenance condition from the theta power ratio of the regulation condition for each participant.

In this analysis, a comprehensive whole-brain comparison was conducted to investigate differences in theta power ratio changes, employing a two-sided hypothesis test with a significance threshold set at an alpha level of 0.05. Determination of statistical significance for the observed differences was achieved through a permutation-based approach, starting with calculating the mean theta power ratio difference between the two groups for each voxel. This was followed by 100 000 permutations, wherein each permutation involved randomly reassigning the group labels of the data and recalculating the mean differences for these newly formed groups. This process generated a distribution of mean differences under the null hypothesis, which assumes no actual difference between the groups.

*P*-values were derived from this distribution by determining the proportion of permutations in which the recalculated mean difference was as extreme as or more extreme than the observed mean difference for each voxel. Adjusting this proportion to account for the total number of permutations provided the *P*-value for each voxel.

### Multivariate interaction measure (MIM)

Whole-brain functional connectivity was analysed by calculating the multivariate interaction measure (MIM) within the theta frequency band (3.5–8.5 Hz) on the pre-processed single-trial data.^32^ MIM is a coupling measure robust to artifacts of volume conduction, i.e. it vanishes for a linear mixture of independent sources, which otherwise causes a severe problem for coupling analysis not only at the sensor level but also at the source level because inverse calculations are not unique and cannot avoid mixing of true brain sources. MIM is designed to address the multivariate nature of estimated source activities in a voxel, i.e. it is a three-dimensional signal corresponding to three source directions. MIM calculates the coupling between two voxels in a way that is invariant to rotations of source orientations for each voxel. It can be expressed as a sum of three eigenvalues, reducing the interaction measure to a single value per frequency band and pair of voxels. The largest eigenvalue here is the coupling corresponding to those source orientations that maximise the coupling for each given pair of voxels (the other eigenvalues contain additional orthogonality constraints analogous to a typical principal component analysis decomposition). This approach should be contrasted with alternatives in which the source direction is assumed to be fixed, for example by maximising power for each voxel. These alternatives can miss relevant coupling because strong sources are not necessarily the ones with strong coupling.

For this study, MIM was applied to assess interactions between individual voxels and the entire cortex. Connectivity for each voxel was calculated by averaging its MIM values with those of all other voxels, providing a detailed view of its connectivity with the whole cortex.

It is important to note that MIM effectively measures interactions between distinct brain areas, rather than within them. Particularly, in our voxel-to-whole-brain connectivity analysis, where we examined how each voxel connects on average to all other voxels on the cortex surface, this characteristic of MIM is pivotal. Significant findings in this context imply that a particular voxel has notable connections to other regions across the cortex. These connections are not due to internal changes or mixed activities within the voxel itself, but rather represent true interactions between that voxel and the broader brain network. Such results are key to understanding the role of specific brain areas in overall brain function and connectivity.

The statistical approach for analysing functional connectivity differences mirrored that employed for source power analysis.

### Statistics

All statistical analyses were performed in Matlab (MathWorks® for Windows). Differences between groups with respect to the demographic data, interpolated electrodes and ICA components were assessed with independent-sample *t*-tests. The differences in number of trials and the ratings of emotional responses between the groups and the conditions (within-participant) were tested with a mixed-model two-way analysis of variance (ANOVA). When significant interaction effects (group × condition) were observed, we conducted Bonferroni-corrected simple-effects analyses. In the absence of significant interactions, we focused on analysing the main effects. The differences in ERQ scores were analysed in a similar way, using the ERQ facet scores Cognitive Reappraisal (‘Reappraisal’) and Expressive Suppression (‘Suppression’) as the within-participant factors.

The prediction of regulation-associated theta activity differences via ERQ scores and behavioural ratings was tested with a multiple linear regression.

Statistical significance was set at an alpha level of 0.05 for all analyses.

## Results

### Behavioural results

Both groups reported less negative emotions in the reappraisal condition compared with the maintenance condition, as indicated by a significant condition effect (*F*_1,94_ = 28.7, *P* < 0.001). Specifically, participants with BPD reported lower (*P*_adjusted_ = 0.007) negative emotion ratings in the reappraisal condition (mean 42, s.d. = 21) than in the maintenance condition (mean 58, s.d. = 25). In comparison, the matched control group reported slightly lower ratings in the reappraisal condition (mean 35, s.d. = 19), showing a similar trend of reduced (*P*_adjusted_ < 0.001) negative emotion ratings compared with the maintenance condition (mean 56, s.d. = 21). We did not observe significant differences between groups depending on the condition (interaction term; *F*_1,94_ = 0.62, *P* = 0.43) or between groups (*F*_1,94_ = 1.04, *P* = 0.31).

### ERQ rating

The evaluation of the ERQ ratings indicated a significant interaction effect between groups and specific questionnaire facets (F_1,96_ = 15.2, *P* < 0.001). The follow-up simple effects analysis uncovered that participants with BPD had notably lower (*P*_adjusted_ = 0.006) Reappraisal scores (mean 21, s.d. = 8) than matched controls (mean 27, s.d. = 7). Conversely, these participants reported higher (*P*_adjusted_ = 0.048) Suppression scores (mean 15, s.d. = 5) compared with their matched controls (mean 12, s.d. = 5).

### Sensor-level theta power

**Fig. 2:**
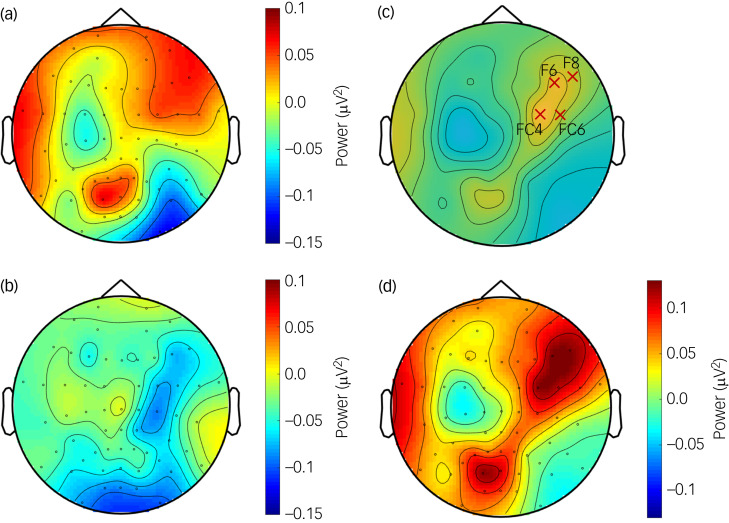
Regulation-associated (reappraisal condition minus maintenance condition) theta activity (3.5–8.5 Hz) following the regulation instruction (1–5 s) in (a) matched controls and (b) women with borderline personality disorder (BPD). (c) Regions showing statistical differences between the two groups (electrodes marked by ×). (d) Differences between the two groups shown by the contrast (controls minus BPD cohort) of topographical activity.

The cluster-based permutation test showed a significantly higher increase in regulation-associated theta activity (reappraisal condition minus maintenance condition) in matched controls than in members of the BPD cohort. This difference was observed in right frontocentral sensors (FC4, FC6, F6 and F8; *P*_cluster_ = 0.039; Fig. 2(c)). Essentially, during cognitive reappraisal, matched controls displayed a more pronounced elevation in theta activity from the maintenance condition compared with the BPD cohort, particularly in these frontocentral regions. The follow-up individual analysis within both the matched controls and the BPD cohort, however, revealed no significant differences between the reappraisal and maintenance conditions.

Multiple linear regression was used to predict the differences in theta activity of the significant cluster between the reappraisal condition and the maintenance condition based on the ERQ Reappraisal and Suppression facet scores as well as the differences of emotional ratings.

**Fig. 3:**
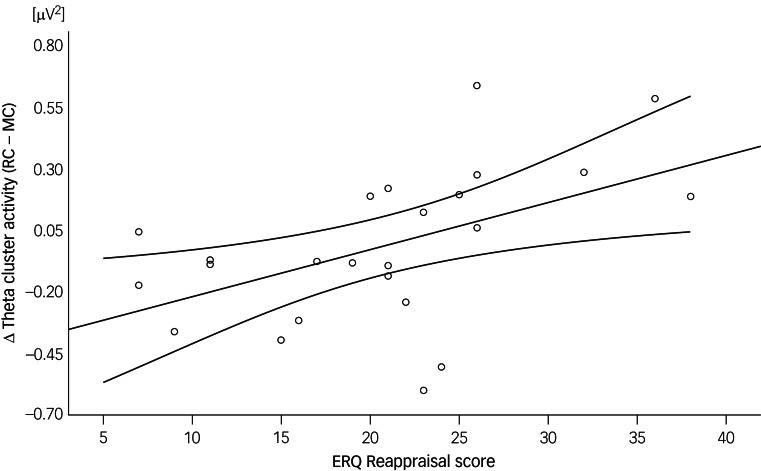
Scores on the Cognitive Reappraisal facet of the self-rated Emotion Regulation Questionnaire (ERQ) and their relation to regulation-associated theta activity in women with borderline personality disorder. RC, reappraisal condition; MC, maintenance condition.

In our analysis of regulation-associated theta activity in the BPD cohort, the overall multiple linear regression model approached but did not reach statistical significance (*F*_3,20_ = 3.04, *P* = 0.053, *R*² = 0.31). In an explorative approach, we further examined the contributions of individual predictors within this model. Notably, the ERQ Reappraisal score emerged as a significant predictor after conducting a Bonferroni correction for multiple comparisons (*t*_20_ = 2.68, *P*_adjusted_ = 0.02; Fig. 3). Specifically, each unit increase in the Reappraisal score was associated with an increase of 0.02 μV² in regulation-associated theta activity. In contrast, the other predictors in the model, namely the ERQ Suppression score (*t*_20_ = −0.47, *P*_adjusted_ = 1) and the differences in emotional ratings (*t*_20_ = −1.1, *P*_adjusted_ = 0.59), did not show significant contributions.

No significant regression equation was found for matched controls (*F*_3,21_ = 0.75, *P* = 0.48).

### Theta source power

**Fig. 4:**
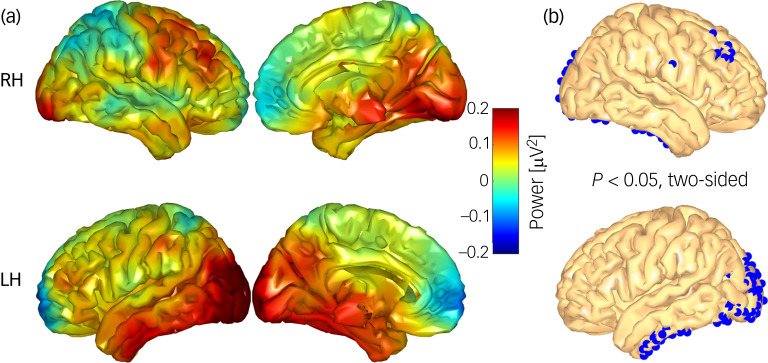
(a) Comparative difference map displaying regulation-associated brain activity between matched controls and women with borderline personality disorder (BPD). (b) Areas (blue voxels) where matched controls exhibited significantly higher source activity compared with the BPD cohort. LH, left hemisphere; RH, right hemisphere.

The voxel-wise whole-brain comparison of the theta-power ratio differences (reappraisal condition minus maintenance condition) revealed a larger increase of theta source activity in the control group compared with the BPD group in three clusters (Fig. 4): a large cluster in the left occipital regions, a cluster of nine voxels in the right dlPFC (Montreal Neurological Institute (MNI) coordinates of the cluster centrum: *x* = 41, *y* = 35, *z* = 40) and a cluster in the left inferior temporal lobe (all *P* < 0.05, two-sided).

**Fig. 5:**
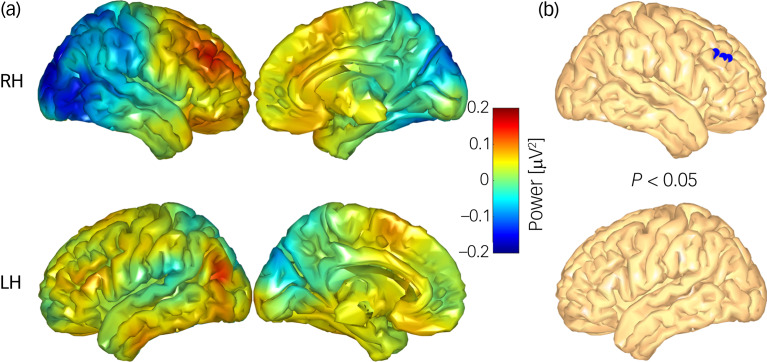
(a) Regulation-associated source activity (reappraisal condition minus maintenance condition) in the control cohort. (b) Areas (blue voxels) where controls exhibited significantly higher source activity in the reappraisal condition compared with the maintenance condition. LH, left hemisphere; RH, right hemisphere).

Follow-up analyses revealed no significant within-group differences, but based on our previous results, we performed a one-tailed test (cut-off level *P* < 0.05) and found a significant increase in theta source power in a cluster of seven voxels in the right dlPFC in the control group (cluster centrum: *x* = 42, *y* = 34, *z* = 35; Fig. 5). No differences were found for the BPD group.

### Theta functional connectivity

**Fig. 6:**
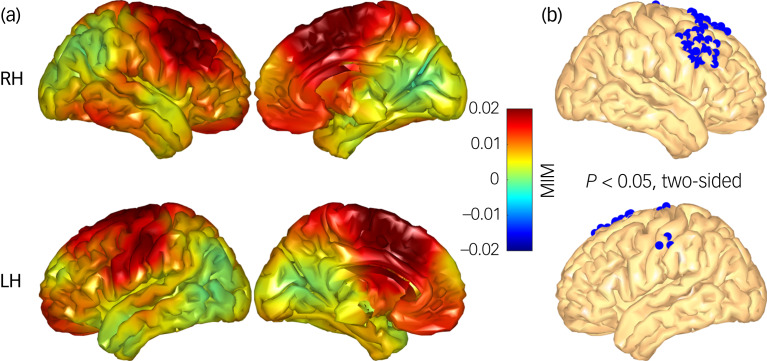
(a) Comparative difference map displaying regulation-associated functional connectivity between matched controls and women with borderline personality disorder (BPD). (b) Areas (blue voxels) where matched controls exhibited significantly higher voxel-to-whole-brain functional connectivity compared with the BPD cohort. LH, left hemisphere; RH, right hemisphere; MIM, multivariate interaction measure.

The voxel-wise comparison of regulation-associated changes of whole-brain functional connectivity (reappraisal condition minus maintenance condition) revealed significantly larger MIM increases in the control group compared with the BPD group in three clusters (Fig. 6): a large cluster in the middle frontal gyrus comprising 39 voxels in the right dlPFC and frontal eye fields (cluster centrum: *x* = 47, *y* = 17, *z* = 43), a large cluster in the superior motor cortex and a small cluster in the left primary somatosensory cortex (all *P* < 0.05, two-sided).

## Discussion

In the present study, we investigated oscillatory brain dynamics during the regulation of emotions by cognitive reappraisal to determine whether there are differences between women diagnosed with BPD and matched controls in the coupling of theta oscillations in frontal brain regions. In line with our hypotheses, we found significant differences in the theta band (3.5–8.5 Hz) between patients and matched controls. Our analyses further revealed that during reappraisal oscillatory theta activity was increased in a frontal cluster within the right dlPFC, but only in the matched control sample. The group comparison revealed a significantly lower increase of theta activity within this dlPFC cluster during reappraisal in the participants with BPD. Nonetheless, the self-rated emotion regulation strategy score for reappraisal was found to be significantly positively associated with reappraisal-associated differences in theta activity in the BPD cohort. Analyses of regulation-associated changes in functional connectivity in the theta frequency range revealed a reduced connectivity between a cluster encompassing the right dlPFC cluster and all other cortex regions in the participants with BPD.

Previous research on theta oscillatory activity in the downregulation of negative emotions among healthy individuals has consistently shown an increase in this activity, albeit with variations in timing and spatial distribution.^16–18^ These studies have explored various regulation strategies, such as cognitive reappraisal, distancing and distraction. Notably, both distraction^17^ and distancing^18^ are associated with an early surge in theta activity taking place in the first second within frontal regions, likely linked to an initial, automatic assessment of the emotional stimulus. In contrast, cognitive reappraisal,^16^ a more complex strategy, involves a self-relevant reinterpretation of the emotional stimulus and engages various regions within a frontoparietal executive network^7^ during later processing stages (1–5 s). In our study, although a comparable increase in theta activity in frontal areas during reappraisal was observed, we did not find a correlation between this activity and regulatory success in healthy controls, as reported by Ertl et al.^16^ This divergence could be due to variations in experimental design and statistical approaches.

One mechanism common to the various approaches is that different brain areas most likely have to interact with each other in the application of emotion regulation strategies.^14^ Neuroimaging studies have demonstrated increased activity in prefrontal areas, as well as decreased amygdala activity during the application of downregulating strategies.^6^ Accordingly, animal studies using fear conditioning and extinction paradigms show that there is increased coupling in the theta band between prefrontal areas and subcortical areas such as the amygdala.^15,33^ Taken together with previous results on the role of theta in the early processing of emotion, we propose theta dynamics as a marker for different stages of emotion regulation.

Previous findings on the neural correlates of emotion regulation in individuals diagnosed with BPD point to an aberrant frontolimbic inhibitory network underlying the inter- and intrapersonal emotional deficiencies.^11,12,21^ Yet, to our knowledge no prior study has reported on emotion regulation-associated oscillatory activity in people diagnosed with BPD in comparison with volunteers without a history of receiving psychiatric care. Using source localisation, our results revealed larger differences in theta source activity in the control group compared with the BPD group in a large cluster in the left occipital regions, a cluster in the right middle frontal gyrus and a cluster in the left inferior temporal lobe. The individual analysis for each group highlighted a significant increase of theta source power in the right middle frontal gyrus in the reappraisal condition compared with the maintenance condition for the matched controls. However, this difference could not be seen in the BPD cohort. In line with previous neuroimaging studies, the observed differences may be indicative of aberrant activity of a frontoparietal network in BPD, which has been linked to successful cognitive reappraisal.^6,7^

Further, analysis of functional connectivity for regulation-associated changes in the theta frequency band revealed a significant increase in the multivariate interaction measure (MIM) in the frontal brain areas in controls compared with the BPD sample. As reappraisal success in the downregulation of emotion has been linked to effective connectivity between frontal brain regions associated with cognitive control, such as the inferior frontal gyrus and dorsal prefrontal regions,^34^ our results support the notion of diverging frontal brain activity in individuals diagnosed with BPD.^9–11^

Our findings add to previous neuroimaging studies showing that the downregulation of negative emotions is associated with coupling in the theta band between prefrontal regions and the amygdala.^7^ Consistent with previous studies of increased frontal theta oscillations during cognitive reappraisal in individuals without a history of receiving psychiatric care^16^ and of distinct connectivity patterns underlying mental disorders characterized by challenges in emotion regulation,^34^ our findings reveal specific, differing theta dynamics in BPD. In this context, our results may help to clarify altered oscillatory dynamics in psychiatric disorders and thereby provide a basis for future research on new treatment strategies.

## Limitations and prospective studies

In this study, the participants’ initial emotional responses to the images, prior to their engagement in emotion regulation strategies, were not assessed. Thus, regulatory success for each stimulus was not measured directly, and therefore results on regulatory success should be treated with caution. Additionally, the non-significant overall regression model for the BPD cohort (*P* = 0.053) and reliance on individual predictors such as the ERQ Reappraisal score necessitate cautious interpretation, especially given our study's small sample size. These results underscore the need for further research with larger samples and varied methodologies to robustly delineate the role of specific emotion regulation strategies in individuals with BPD. Moreover, the small sample size of our study may explain the missing correlation between regulatory success and theta performance at the sensor level in the control group. Owing to the amygdala's subcortical location, EEG-based source localisation cannot directly measure its activity or its interactions with prefrontal areas. This limitation is compounded by the use self-reported emotion regulation success as a proxy for amygdala activation, given the variability and subjective nature of self-reported measures. Individuals’ self-reports can vary widely and be influenced by factors such as introspective accuracy and social desirability, which may not consistently reflect actual amygdala activation patterns.

Given the low spatial resolution of EEG and the potential drawbacks of indirect estimates of limbic activity, future studies could employ the method of simultaneous EEG–fMRI.^35^ EEG-informed fMRI analyses provide the possibility to investigate oscillatory networks comprising subcortical structures.^35^ This approach might allow more precise recordings of the synchronisation in the theta band during emotion regulation involving subcortical limbic structures.

Interesting new approaches to modulate neurodynamics in psychiatric conditions include transcranial direct current stimulation (tDCS) and transcranial alternating current stimulation (tACS).^36^ For example, enhancing dlPFC excitability through tDCS led to a more successful downregulation of negative emotions by cognitive reappraisal.^37^ By altering varying brain oscillations, tACS has been suggested to be a potentially effective method without severe side-effects in the treatment of various psychiatric disorders, according to a recent review.^38^ Our results suggest the application of tACS to target emotion regulation deficits in a spatially and frequency-adapted individualised manner.

## Supporting information

Haaf et al. supplementary material Haaf et al. supplementary material

## Data Availability

The data presented in this study are available on request from the corresponding author, M.H. The data are not publicly available owing to privacy restrictions and require approval of a data-sharing agreement.

## References

[1] Lenzenweger MF, Lane MC, Loranger AW, Kessler RC. DSM-IV personality disorders in the national comorbidity survey replication. Biol Psychiatry 2007; 62: 553–64. 17217923 10.1016/j.biopsych.2006.09.019 PMC2044500

[2] Carpenter RW, Trull TJ. Components of emotion dysregulation in borderline personality disorder: a review. Curr Psychiatry Rep 2013; 15(1): 335. 23250816 10.1007/s11920-012-0335-2 PMC3973423

[3] Crowell SE, Beauchaine TP, Linehan MM. A biosocial developmental model of borderline personality: elaborating and extending Linehan's theory. Psychol Bull 2009; 135(3): 495–510. 19379027 10.1037/a0015616 PMC2696274

[4] Ntshingila N, Poggenpoel M, Myburgh CPH, Temane A. Experiences of women living with borderline personality disorder. Health Sa Gesondheid 2016; 21: 110–9.

[5] Phillips ML, Drevets WC, Rauch SL, Lane R. Neurobiology of emotion perception I: the neural basis of normal emotion perception. Biol Psychiatry 2003; 54: 504–14. 12946879 10.1016/s0006-3223(03)00168-9

[6] Buhle JT, Silvers JA, Wager TD, Lopez R, Onyemekwu C, Kober H, et al. Cognitive reappraisal of emotion: a meta-analysis of human neuroimaging studies. Cereb Cortex 2013; 24: 2981–90. 23765157 10.1093/cercor/bht154 PMC4193464

[7] Etkin A, Büchel C, Gross JJ. The neural bases of emotion regulation. Nat Rev Neurosci 2015; 16: 693–700. 26481098 10.1038/nrn4044

[8] Berboth S, Morawetz C. Amygdala-prefrontal connectivity during emotion regulation: a meta-analysis of psychophysiological interactions. Neuropsychologia 2021; 153: 107767. 33516732 10.1016/j.neuropsychologia.2021.107767

[9] Krause-Utz A, Winter D, Niedtfeld I, Schmahl C. The latest neuroimaging findings in borderline personality disorder. Curr Psychiatry Rep 2014; 16(3): 438. 24492919 10.1007/s11920-014-0438-z

[10] Schulze L, Schmahl C, Niedtfeld I. Neural correlates of disturbed emotion processing in borderline personality disorder: a multimodal meta-analysis. Biol Psychiatry 2016; 79: 97–106. 25935068 10.1016/j.biopsych.2015.03.027

[11] Lapomarda G, Grecucci A, Messina I, Pappaianni E, Dadomo H. Common and different gray and white matter alterations in bipolar and borderline personality disorder: a source-based morphometry study. Brain Res 2021; 1762: 147401. 33675742 10.1016/j.brainres.2021.147401

[12] Schulze L, Domes G, Krüger A, Berger C, Fleischer M, Prehn K, et al. Neuronal correlates of cognitive reappraisal in borderline patients with affective instability. Biol Psychiatry 2011; 69: 564–73. 21195392 10.1016/j.biopsych.2010.10.025

[13] Mulert C, Jager L, Schmitt R, Bussfeld P, Pogarell O, Moller HJ, et al. Integration of fMRI and simultaneous EEG: towards a comprehensive understanding of localization and time-course of brain activity in target detection. Neuroimage 2004; 22: 83–94. 15109999 10.1016/j.neuroimage.2003.10.051

[14] Sauseng P, Klimesch W. What does phase information of oscillatory brain activity tell US about cognitive processes? Neurosci Biobehav Rev 2008; 32: 1001–13. 18499256 10.1016/j.neubiorev.2008.03.014

[15] Lesting J, Narayanan RT, Kluge C, Sangha S, Seidenbecher T, Pape HC. Patterns of coupled theta activity in amygdala-hippocampal-prefrontal cortical circuits during fear extinction. PLoS One 2011; 6(6): e21714. 21738775 10.1371/journal.pone.0021714 PMC3125298

[16] Ertl M, Hildebrandt M, Ourina K, Leicht G, Mulert C. Emotion regulation by cognitive reappraisal – the role of frontal theta oscillations. Neuroimage 2013; 81: 412–21. 23689018 10.1016/j.neuroimage.2013.05.044

[17] Uusberg A, Thiruchselvam R, Gross JJ. Using distraction to regulate emotion: insights from EEG theta dynamics. Int J Psychophysiol 2014; 91: 254–60. 24440597 10.1016/j.ijpsycho.2014.01.006

[18] Sulpizio S, Grecucci A, Job R. Tune in to the right frequency: theta changes when distancing from emotions elicited by unpleasant images and words. Eur J Neurosci 2021; 53: 916–28. 33091188 10.1111/ejn.15013

[19] Korotkova T, Ponomarenko A, Monaghan CK, Poulter SL, Cacucci F, Wills T, et al. Reconciling the different faces of hippocampal theta: the role of theta oscillations in cognitive, emotional and innate behaviors. Neurosci Biobehav Rev 2018; 85: 65–80. 28887226 10.1016/j.neubiorev.2017.09.004

[20] Hallam GP, Webb TL, Sheeran P, Miles E, Wilkinson ID, Hunter MD, et al. The neural correlates of emotion regulation by implementation intentions. PLoS One 2015; 10(3): e0119500. 25798822 10.1371/journal.pone.0119500 PMC4370584

[21] Sicorello M, Schmahl C. Emotion dysregulation in borderline personality disorder: a fronto-limbic imbalance? Curr Opin Psychol 2021; 37: 114–20. 33422855 10.1016/j.copsyc.2020.12.002

[22] First MB, Spitzer RL, Gibbon M, Williams BW, Benjamin L. Structured Clinical Interview for DSM-IV Axis II Personality Disorders (SCID-II). Biometrics Research Department, New York State Psychiatric Institute, 1990.

[23] Montgomery SA, Asberg M. A new depression scale designed to be sensitive to change. Br J Psychiatry 1979; 134: 382–9. 444788 10.1192/bjp.134.4.382

[24] Lang PJ, Bradley MM, Cuthbert BN. *International Affective Picture System (IAPS): Affective Ratings of Pictures and Instruction Manual*. Technical Report A-8. University of Florida, 2008.

[25] Gross JJ. Antecedent- and response-focused emotion regulation: divergent consequences for experience, expression, and physiology. J Pers Soc Psychol 1998; 74: 224–37. 9457784 10.1037//0022-3514.74.1.224

[26] Gross JJ. Emotion regulation in adulthood: timing is everything. Curr Dir Psychol Sci 2001; 10: 214–9.

[27] Gross JJ, John OP. Individual differences in two emotion regulation processes: implications for affect, relationships, and well-being. J Pers Soc Psychol 2003; 85: 348–62. 12916575 10.1037/0022-3514.85.2.348

[28] Abler B, Kessler H. Emotion Regulation Questionnaire – eine deutschsprachige Fassung des ERQ von Gross und John (Emotion Regulation Questionnaire – A German version of the ERQ by Gross and John). Diagnostica 2009; 55: 144–52.

[29] Oldfield RC. The assessment and analysis of handedness: the Edinburgh Inventory. Neuropsychologia 1971; 9: 97–113. 5146491 10.1016/0028-3932(71)90067-4

[30] Oostenveld R, Fries P, Maris E, Schoffelen JM. Fieldtrip: open source software for advanced analysis of MEG, EEG, and invasive electrophysiological data. Comput Intell Neurosci 2011; 2011: 156869. 21253357 10.1155/2011/156869 PMC3021840

[31] Pascual-Marqui RD, Lehmann D, Koukkou M, Kochi K, Anderer P, Saletu B, et al. Assessing interactions in the brain with exact low-resolution electromagnetic tomography. Philos Trans A Math Phys Eng Sci 2011; 369: 3768–84. 21893527 10.1098/rsta.2011.0081

[32] Ewald A, Marzetti L, Zappasodi F, Meinecke FC, Nolte G. Estimating true brain connectivity from EEG/MEG data invariant to linear and static transformations in sensor space. Neuroimage 2012; 60: 476–88. 22178298 10.1016/j.neuroimage.2011.11.084

[33] Chen S, Tan Z, Xia W, Gomes CA, Zhang X, Zhou W, et al. Theta oscillations synchronize human medial prefrontal cortex and amygdala during fear learning. Sci Adv 2021; 7(34): eabf4198. 34407939 10.1126/sciadv.abf4198 PMC8373137

[34] Morawetz C, Bode S, Baudewig J, Heekeren HR. Effective amygdala-prefrontal connectivity predicts individual differences in successful emotion regulation. Soc Cogn Affect Neurosci 2017; 12: 569–85. 27998996 10.1093/scan/nsw169 PMC5390747

[35] Mulert C, Leicht G, Hepp P, Kirsch V, Karch S, Pogarell O, et al. Single-trial coupling of the gamma-band response and the corresponding BOLD signal. Neuroimage 2010; 49: 2238–47. 19878729 10.1016/j.neuroimage.2009.10.058

[36] Polanía R, Nitsche MA, Ruff CC. Studying and modifying brain function with non-invasive brain stimulation. Nat Neurosci 2018; 21: 174–87. 29311747 10.1038/s41593-017-0054-4

[37] Feeser M, Prehn K, Kazzer P, Mungee A, Bajbouj M. Transcranial direct current stimulation enhances cognitive control during emotion regulation. Brain Stimul 2014; 7: 105–12. 24095257 10.1016/j.brs.2013.08.006

[38] Elyamany O, Leicht G, Herrmann CS, Mulert C. Transcranial alternating current stimulation (tACS): from basic mechanisms towards first applications in psychiatry. Eur Arch Psychiatry Clin Neurosci 2021; 271: 135–56. 33211157 10.1007/s00406-020-01209-9 PMC7867505

