# Supplementary material for: Frontal theta oscillations during emotion regulation in people with borderline personality disorder
Source: BJPsych Open. 2024 Mar 4;10(2):e58. doi: 10.1192/bjo.2024.17 (PMC10951849; doi:10.1192/bjo.2024.17)
Supplement: Haaf et al. supplementary material [file S2056472424000176sup001.docx]

Supplemental Information

# Supplemental Methods

## Emotion regulation strategy

When asked to counteract negative reactions to a picture of an individual undergoing open-heart surgery, participants should imagine that the patient is resilient, or that this operation is precautionary, or that the operation will greatly help the patient in the long run. Furthermore, they were also told, that they could reappraise a negative emotional response by imagining that a sick person will soon get better, that a gruesome scene is faked (e.g., staged or a prank), or that a scary scene has a positive ending. Participants were instructed to remain within the context of the scene and not to change their emotional response by thinking of a personal memory or of a situation which is not related to the given scene. Participants were asked to try out different strategies using the training set in order to find the best individual strategy.

## Stimuli

Two sets of negative pictures (50 each) were created and balanced based on their valence (both sets: 2.2 ± 0.4) and arousal (set 1: 6 ± 0.7; set 2: 5.9 ± 0.7) values for female subjects (neutral set: valence 5 ± 0.3; arousal 3.1 ± 0.5). A training set comprised 11 negative and 6 neutral pictures.

## Paradigm

The trial started with a black screen presented for a duration of 500 ms followed by presentation of a fixation cross for a duration of 2.5 seconds. The fixation cross was used in order to reduce [eye-movements](https://www.sciencedirect.com/topics/neuroscience/eye-movements) and focus the gaze at the center of the screen for picture onset. Thereafter, the picture was presented for 10 seconds. Three seconds after picture onset a digitized human voice gave a one-word instruction “verringern” (engl. decrease) or “fortfahren” (engl. maintain), marking the beginning of the emotion regulation phase of the trial. Thereafter, a visual emotion rating scale was presented with extremes marked with "negative" (max. rating value 100) and "neutral" (min. rating value 0). The participants were instructed to spontaneously rate their present emotion by moving a bar on the scale with a computer mouse in their hands. After pressing the mouse button, the word "Relax" appeared on the screen for two seconds and the trial ended.

The order of pictures within the conditions was randomized for each participant at the beginning of the experiment. After data collection from half of the participants in each group, the negative images were swapped between MC and RC to eliminate a confounding effect of the image content.

## EEG acquisition

Electrodes were positioned in an extended 10/20 system with the additional positions: AF7, AF3, AF4, AF8, F5, F1, F2, F6, F10, FT9, FT7, FC3, FC4, FT8, FT10, C5, C1, C2, C6, TP7, CPz, TP8, P5, P1, P2, P6, PO3, POz, PO4. All electrodes were referenced during recording to FCz and AFz served as ground.

Data were recorded with a bandpass filter (0.1–1000 Hz) and digitized at a sampling rate of 1000 Hz.

## EEG preprocessing

The data was high-pass (100 Hz, order 8, padding with data to 15 s) and low-pass filtered (0.3 Hz, order 4, padding with data to 15 s) employing Butterworth filter. For the interpolation of noisy channels, a 4th order spherical spline interpolation (Perrin et al., 1989) using a regularization parameter lambda of 1e-5 was employed.

The independent component analysis was based on an infomax extended ICA algorithm with PCA dimension reduction for interpolated electrodes (stop criterion: weight change 10^-7^) (Makeig et al., 1996).

## eLORETA source localization

The fast Fourier transformation was performed using a Hanning Taper with 0.5 Hz steps from 3.5–8.5 Hz (with data zero padded to the onset of the regulation instruction at 5 s).

eLORETA is a discrete, linear, three-dimensional distributed, weighted minimum norm inverse solution, which localizes the power distribution of the EEG signal with exact maxima for single dipoles but with low spatial resolution (Pascual-Marqui et al., 2011).

# Supplemental results

## EEG preprocessing

The mean number of interpolated channels did not significantly differ between groups (healthy participants: 6 ± 2 [mean ± SD]; patients: 7 ± 2; t[48] = 0.80, p=0.43).

There was neither a significant interaction effect between groups and conditions [F[1, 48] = 0.03, p = 0.87] nor a significant difference between conditions (F[1, 48] = 3.1, p = 0.1) with respect to the number of trials used for averaging. The trial count differed between groups (healthy participants: 31 ± 5.7 in MC and 32.4 ± 6.4 in RC; patients: 27.5 ± 6.4 in MC and 28.6 ± 6.3 in RC; F[1, 48] = 5.3, p = 0.026).

There was no significant difference between groups with respect to the number of removed components (healthy participants: 4.0 ± 1.3; patients: 3.4 ± 1.2; t[48] = 1.5, p = 0.14).

# Supplemental references

Makeig, S., Bell, A. J., Jung, T. P., & Sejnowski, T. J. (1996). Independent component analysis of electroencephalographic data. *Advances in Neural Information Processing Systems*, *8*, 145--151.

Pascual-Marqui, R. D., Lehmann, D., Koukkou, M., Kochi, K., Anderer, P., Saletu, B., Tanaka, H., Hirata, K., John, E. R., Prichep, L., Biscay-Lirio, R., & Kinoshita, T. (2011). Assessing interactions in the brain with exact low-resolution electromagnetic tomography. *Philos Trans A Math Phys Eng Sci*, *369*(1952), 3768-3784. <https://doi.org/10.1098/rsta.2011.0081>

Perrin, F., Pernier, J., Bertrand, O., & Echallier, J. F. (1989). Spherical splines for scalp potential and current density mapping. *Electroencephalography and Clinical Neurophysiology*, *72*(2), 184-187. <https://doi.org/0013-4694(89)90180-6> [pii]
